# Monte Carlo calculations of output factors for clinically shaped electron fields

**DOI:** 10.1120/jacmp.v5i2.1976

**Published:** 2004-08-16

**Authors:** Julius V. Turian, Brett D. Smith, Damian A. Bernard, Katherine L. Griem, James C. Chu

**Affiliations:** ^1^ Department of Radiation Oncology University of Illinois Medical Center OCC C‐400 1801 W. Taylor Street Chicago Illinois 60612 U.S.A.; ^2^ Department of Radiation Oncology Rush Medical Center 1635 West Congress Parkway Chicago Illinois 60612 U.S.A.

**Keywords:** output factors, electron beams, Monte Carlo simulation

## Abstract

We report on the use of the EGS4/BEAM Monte Carlo technique to predict the output factors for clinically relevant, irregularly shaped inserts as they intercept a linear accelerator's electron beams. The output factor for a particular combination—energy, cone, insert, and source‐to‐surface distance (SSD)—is defined in accordance with AAPM TG‐25 as the product of cone correction factor and insert correction factor, evaluated at the depth of maximum dose. Since cone correction factors are easily obtained, we focus our investigation on the insert correction factors (ICFs). An analysis of the inserts used in routine clinical practice resulted in the identification of a set of seven “idealized” shapes characterized by specific parameters. The ICFs for these shapes were calculated using a Monte Carlo method (EGS4/BEAM) and measured for a subset of them using an ion chamber and well‐established measurement methods. Analytical models were developed to predict the Monte Carlo–calculated ICF values for various electron energies, cone sizes, shapes, and SSDs. The goodness‐of‐fit between predicted and Monte Carlo–calculated ICF values was tested using the Kolmogorov–Smirnoff statistical test. Results show that Monte Carlo–calculated ICFs match the measured values within 2.0% for most of the shapes considered, except for few highly elongated fields, where deviations up to 4.0% were recorded. Predicted values based on analytical modeling agree with measured ICF values within 2% to 3% for all configurations. We conclude that the predicted ICF values based on modeling of Monte Carlo–calculated values could be introduced in clinical use.

PACS numbers: 87.53.Wz, 87.53.Hv

## I. INTRODUCTION

Electron beams are frequently used in radiotherapy for the treatment of superficial lesions. Due to the omnipresence of the scattering effects, the dosimetry of these beams depends strongly on the energy, collimation, and geometry layout of the medium transversed by the electrons. While it is straightforward to predict the output factors (OFs, in cGy/MU) for regular fields, complications arise when a unified approach is attempted for the irregular fields. The large variety of energies, field sizes, field shapes, and treatment distances (source‐to‐surface distances, SSD) used in clinical practice is an impediment in creating a standard model, and quite frequently the clinical medical physicist has to resort to measuring the OF for a particular configuration. Moreover, OF measurements, especially for small fields, are known to be prone to errors, since the dose distribution is not uniform at the point of measurement on the central axis. Therefore, accurate methods of predicting and measuring the OFs for electron beams need to be available to the clinical physicist.

Considerable effort^(^ ^1^ ^–^ ^8^ ^)^ has been made over the years to improve the ability to predict the OFs for clinical electron beams using empirical analytical models. More recently, a number of investigators^(^ ^9^ ^–^ ^12^ ^)^ have been evaluating Monte Carlo methods to calculate OFs for electron beams shaped using square, rectangular, and circular inserts. The consensus among investigators is that the Monte Carlo methods have the potential to accurately predict the OFs for electron beams.^(13)^ Due to rapid improvements in computer technology, they are fast becoming the standard alternative for obtaining accurate dosimetric quantities for electron beams used in radiotherapy. Currently, OFs for electron beams could easily be calculated for every energy and cutout shape using Monte Carlo methods if the simulation model of the linear accelerator exists. Nevertheless, in clinical practice it is required that accurate OF values be readily available without the need for users to specify the geometry of the field and run the corresponding simulations.

In this study we present a practical method to determine the OFs based on Monte Carlo simulations. The results are verified for a range of insert shapes and electron energies.

## II. MATERIALS AND METHODS

### A. Measurements

All measurements were performed on a Clinac 2100 EX (Varian Medical Systems, Palo Alto, CA) linear accelerator equipped with Type III electron applicators. Central axis percent depth‐dose (%DD) was measured for a range of square field sizes ($3\times 3\;{\text{cm}}^{2}$ to $25\times 25\;{\text{cm}}^{2}$) at 100 cm SSD and for selected fields $\left(15\times 15\;{\text{cm}}^{2}\right)$ at 115 cm SSD. The measurements were made using a 0.0012 cm^3^ active volume (0.25‐cm diameter and 0.006‐cm thickness) p‐type electron diode detector (Model 999‐600 EFD Hi‐*p*Si) in a water phantom (Scanditronix Medical AB, Uppsala, Sweden). Good spatial resolution (~1.0 mm) and the ability to measure the dose directly are good incentives for selecting these detectors. One drawback of the diode measurements was its over‐response to the photon component of the electron beam. It was unclear to us if the over‐response was due to the detector itself or our inability to properly account for the background signal. To correct for this inconsistency, the photon contamination dose component, $D_{x}$, as a percentage of maximum dose, ${\text{DO}}_{\max}$, was measured using the parallel‐plate ion chamber (PTW Markus Model N23343, PTW, Freiburg, Germany) in an electron solid water phantom (Gammex RMI Model 457, Middletown, WI).

Cross‐beam profiles in a plane that contained the central axis were measured for a range of field sizes ($3\times 3\;{\text{cm}}^{2}$ to $25\times 25\;{\text{cm}}^{2}$) at 100‐cm SSD and for selected field sizes $\left(15\times 15\;{\text{cm}}^{2}\right)$ at 115 cm SSD. Cross‐beam profiles were measured at several depths as suggested by the TG‐25^(15)^ report using the p‐type electron diode detector.

Insert correction factors (ICFs) were measured for a given energy/applicator/insert using a Markus parallel‐plate ion chamber in an electron solid water phantom. The active volume of the chamber is 0.05 cm^3^, and the diameter of the window is 0.54 cm, thus making it unsuitable for measurements when the size of the field is very small. Based on the above‐mentioned limitation and clinical experience, the lower limit for the field size was set to 2.0 cm. The measurements were performed at the depth of maximum dose, $R_{100}$, for each insert. In accordance with a report by Zhang et al.,^(16)^ using ionization ratios instead of dose ratios can lead to 3% error in the ICF; therefore, dose ratios were used in this work. The field size effect of the stopping power ratios was ignored, and the mono‐energetic stopping power ratios^(15)^ were employed instead of realistic beam data. The replacement correction factor as a function of depth for the Markus‐type ion chamber was calculated using the formalism described in TG‐39.^(17)^

**Figure 1:**
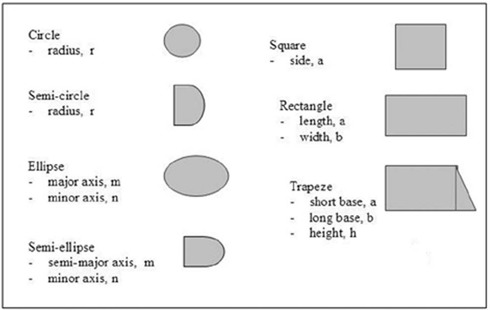
Idealized clinical shapes used for ICF evaluation. Each shape is described by one, two, or three parameters. Modifying the parameters changes the size of the insert but not the shape.

A number of successful attempts were made^(^ ^9^ ^–^ ^12^ ^)^ to predict the values of OFs for a given insert using Monte Carlo methods. Most of the work was done for regular inserts (circle, square, and rectangle). In this work, the insert shapes used frequently in the clinic were investigated and standardized to a set of seven shapes. A list of these shapes and their associated parameters is shown in Fig. 1. For inserts whose shape is different than the ones shown in Fig. 1, an approximation to these shapes can be performed. The inserts for each applicator were made out of Cerrobend, a low‐melting point lead alloy.

### B. Definition

The OF for a specific energy, *E*, applicator size, $C_{s}$, insert size, $I_{s}$, and SSD is defined in TG‐25^(15)^ as

(1) $$\text{OF}\left(E,C_{s},I_{s}\right)={\left(\frac{\frac{D_{\text{max}}}{\text{MU}}\left(E,C_{s},I_{s}\right)}{\frac{D_{\text{max}}}{\text{MU}}(E,C_{0},I_{0})}\right)}_{\text{SSD}=\text{const}}$$

where $D_{\max}/\text{MU}$ is the dose per monitor unit at $R100(R_{100})$; $C_{0}$ is the standard applicator used for dose output calibration, and $I_{0}$ is the open applicator insert unique for any given applicator. Through simple mathematical manipulation, Eq. (1) can be rewritten as

(2) $$\text{OF}\left(E,C_{s},I_{s}\right)=\frac{\frac{D_{\text{max}}}{\text{MU}}\left(E,C_{s},I_{0}\right)}{\frac{D_{\text{max}}}{\text{MU}}(E,C_{0},I_{0})}\times \frac{\frac{D_{\text{max}}}{\text{MU}}(E,C_{s},I_{s})}{\frac{D_{\text{max}}}{\text{MU}}(E,C_{s},I_{0})}=\text{CCF}\times \text{ICF}$$

where the first term, the cone correction factor (CCF), is the dose per monitor unit ratio between applicator size $C_{s}$ and the standard applicator $C_{0}$, and the second term, insert correction factor (ICF), is the dose per monitor unit ratio between insert size $I_{s}$ and the standard insert $I_{0}$ for the same applicator.^(15)^

When measurements are performed using ion chamber detectors, the definition of ICF as represented in Eq. (2) is no longer valid, and additional corrections have to be introduced. The corrections are needed due to the dependence of the average stopping power ratios (L¯ρ)airmed and chamber correction factors, on the depth of measurement. Ignoring these effects can overestimate the measured ICF by up to 3% according to Zhang et al.^(16)^ Since by definition ICF is measured at the depth of maximum dose, $R_{100}$, and $R_{100}$ shifts toward the surface as the size of the field is reduced, one must take into account the depth dependence of the parameters used to compute these factors. The measured ionization is converted to dose by using TG‐21 formalism.^(18)^ For the Markus chamber used in this study $P_{\text{wall}}$ is taken as unity for electron beams in accordance with TG‐21. The dependence of $P_{\text{ion}}$ on field sizes and depth was investigated, and the variation was found to be minimal (range 0.2% to 0.45%). This finding allows for elimination of the $P_{\text{ion}}$ in the formula for ICF (see below). $P_{\text{repl}}$ is unity for well‐guarded plane‐parallel chambers.^(17)^ However, for poorly guarded plane‐parallel chambers, $P_{\text{repl}}$ is a function of depth as is the case for the chamber used in this study. $P_{\text{repl}}$ has been calculated for the Markus chamber using the formalism described in TG‐39.^(17)^

The ICFs are obtained from measurements using the following formula:

(3) ICF(E,Cs,Is)=(DmaxMU(E,Cs,Is,R100)×(L¯ρ)airmed×Prepl|Is,R100DmaxMU(E,Cs,I0,R100,open)×(L¯ρ)airmed×Prepl|I0,R100,open)SSD=const

Ignoring the field size dependence of stopping power ratios will overestimate the ICF by about 0.5 %.^(16)^ In addition, by using mono‐energetic stopping power ratio data instead of realistic beam data. another error of 0.7% is introduced but in the opposite direction. Due to cancelation of these two errors using the data suggested by TG‐25 for mono‐energetic beams and ignoring the field size dependence of the stopping power ratios, the results should be off by not more than 0.4% compared to using realistic beam data and field size dependence. The former approach is used in this study.

### C. Simulations

#### C.1 Monte Carlo simulation model

**Figure 2:**
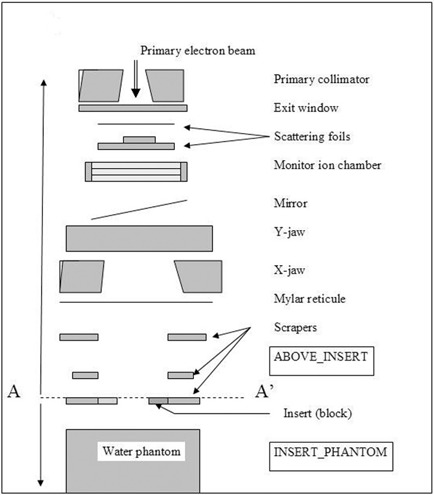
Schematic diagram of the Monte Carlo model used to simulate the Varian Clinac 2100EX electron beams.

**Figure 3:**
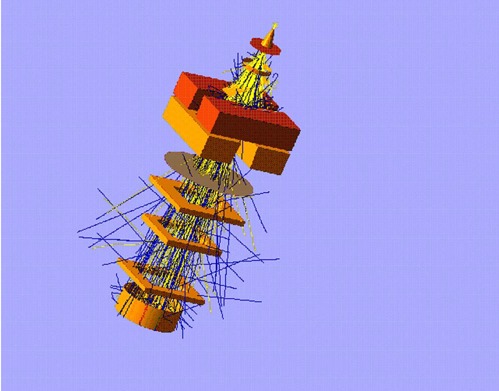
The geometry of the Clinac 2100 EX accelerator head and water phantom used for the tuning phase of the experiment. Electrons are represented by yellow lines and photons by blue lines.

The linear accelerator used in this study, Varian Clinac 2100 EX, was modeled using EGS4/BEAM Monte Carlo code.^(14)^ Figure 2 shows a schematic representation of the linear accelerator head and the components used for simulations. Most of the treatment head geometry and material composition of each component module (CM) was provided by the manufacturer under a confidentiality agreement. The geometry of the standard inserts and Cerrobend blocks was measured directly. The treatment head components included in the model were the tungsten primary collimator, beryllium exit window, tantalum primary scattering foil, aluminum secondary scattering foil, Kapton monitor chamber, aluminized Mylar light mirror, tungsten photon collimator jaws, Mylar reticule, electron applicator (two scrapper bars made out of a zinc alloy and the final field defining insert), and the water phantom. During the first phase (the tuning phase), the entire accelerator head and the water phantom were simulated for all energies and $10\times 10\;{\text{cm}}^{2}$ applicator, and the results used to benchmark the code against measured data. During this phase, the beam was transported from the source to the water phantom. Figure 3 shows the simulation geometry. Depth doses (DDs) can be obtained directly from the output and can be easily compared with measurements. The output was scored at the end of the applicator and the phase space file used as input to BEAM/DOSXYZ^(19)^ to obtain the beam profiles and further analysis.

For the second phase of the project, to avoid redundant calculations, the simulation was divided in two stages. In the first stage, the beam was transported from the source to the top of the insert. The simulation was done once for each energy/applicator combination using the ABOVE_INSERT model, as shown in Fig. 2. These simulations produced phase space files, which were used as input (sources) for the next stage of simulations. In the second stage the beam was transported through the insert and the water phantom. The simulation is done once for every applicator/insert combination using the INSERT_PHANTOM model. All inserts were modeled using BLOCK CM to allow modeling of “irregular” fields. To increase calculation efficiency, the extent of the Cerrobend block outside the aperture was limited to 1.0 mm for 6‐, 9‐, and 12‐MeV beams; 5.0 mm for the 16‐MeV beam, and 30.0 mm for the 20‐MeV electron beam.^(11)^ The number of histories simulated for each instance was chosen to yield a statistical variation of ≤1.0% for the calculated dose. The numbers of histories for stages 1 and 2 were $20\times 10^{6}$ and $40\times 10^{6}$, respectively.

#### C.2 Determination of run parameters

For complete and correct simulations the following quantities must be known: (a) parameters of the primary electron source; (b) the particle's transport parameters, and (c) the size of the scoring voxel in phantom.

The parameters of the electron source include the energy, spatial, and angular distribution. The mean energy of the incident electron beams was chosen so that the calculated and measured half‐value depth in water, $R_{50}$, agreed to within 0.1 cm.^(11)^ Table 1 shows the values of the mean energy used in the simulations for 6‐, 9‐, 12‐, 16‐, and 20‐MeV electron beams. The energy distribution of the primary electron beam was assumed to be mono‐energetic and mono‐directional down the central axis of the treatment head. The spatial distribution of the primary electron beam was considered to be Gaussian in shape with a full‐width‐half‐maximum of 1.5 mm, as described by Jaffray et al.^(20)^ The source described above is available from the BEAM code electron source library.

**Table 1:** Nominal energies $E_{\text{nom}}$, initial electron energies, $E_{i}$, measured $R_{50}$, and calculated $R_{50}$ used for the commissioning phase.

*E*nom (MeV)	$E_{i}$ (MeV)	$R_{50}$(meas) (cm)	$R_{50}$(calc) (cm)	$R_{50}^{calc}-R_{50}^{meas}$
6	7.2	2.42	2.48	0.06
9	10.4	3.47	3.50	0.03
12	13.4	5.01	5.03	0.02
16	17.5	6.65	6.56	–0.09
20	22.9	8.50	8.40	–0.10

The particles' transport model in EGS4 is characterized by four parameters: AE, AP, ECUT, and PCUT. The parameters AE and AP define the threshold energy for the production of secondary electrons and photons, respectively. Low values of AE and AP increase the accuracy of the simulation at the expense of the calculation time. The parameters ECUT (electrons) and PCUT (photons) are cutoff energies for the termination of particle histories. When the energy of the particles falls below the selected values for ECUT and PCUT, the particle deposits its entire energy locally. For all simulations in this study the cutoff kinetic energy for terminating the transport of the electrons was set to ECUT=0.700 MeV(rest+KE), whereas the cutoff energy for terminating the transport of photons was set to PCUT=0.01 MeV. The threshold energy for production of secondary electrons was set to AE=0.700 MeV, whereas the threshold energy for bremsstrahlung creation was set to AP=0.01 MeV. These values where chosen based on work published by other authors^(11)^ ^,^ ^(12)^ ^,^ ^(14)^ and to achieve a compromise between accuracy and speed. Electron range rejection was used for electrons below 2.0 MeV, and the IREJECT_GLOBAL was turned ON either to reject the electrons with insufficient energy to make it to the scoring plane or to reject electrons unable to cross the next boundary. PEGS4 data were consistent with ICRU37^(21)^ data, and the default PRESTA^(22)^ parameters were used for the electron step size.

Depending on the simulation geometry, the output is scored using either the CHAMBER CM as water phantom within BEAM environment or DOSXYZ. The dimensions of the dose scoring voxels strongly influence dose calculation. For large field sizes, where the dose distribution in the center of the field is virtually uniform, the size of the scoring volume is not critical, and large voxel sizes can be used to reduce simulation time while maintaining acceptable uncertainties. For small field sizes the radius of the voxel has to be reduced to minimize the partial fluence averaging effects. Ideally, the dimensions of the scoring regions would be identical to the dimensions of the detector used for the measurements. However, as the scoring volume decreases, the number of required histories increases for a given standard error in the dose. Therefore, it is important to make the dimensions of the scoring volume as large as possible while maintaining acceptable uncertainties for the calculated parameters. In this study, the DDs calculated using CHAMBER CM were scored using a cylindrical voxel of radius r=0.5 cm and height h=0.2 cm for fields smaller than $4\times 4\;{\text{cm}}^{2}$. For fields $\geq 4\times 4\;{\text{cm}}^{2}$, the radius of the voxel was changed to r=1.0 cm while the height was kept constant at 0.2 cm. The agreement between measured and calculated DD was verified using the γ index as described by Low et al.^(23)^ The dose‐difference criterion $\Delta D_{m}$ was set to 2.0%, while the distance to agreement (DTA) criterion, $\Delta d_{m}$, was set to 2.0 mm. The analysis is greatly simplified if the geometrical location of the measurement and calculation are identical.

For instances where cross profiles are needed, the output is scored just below the last scraper and the phase space (phsp) file used as the input to the DOSXYZ code.^(19)^ A scoring grid $v_{x},v_{y},v_{z}$ is set up to generate the profiles. In this study,$v_{x}$ is set along the direction of the profile with dimensions ranging from 0.2 cm to 1.0 cm, $v_{y}=1.0\;\text{cm}$ is set perpendicular to the direction of the profile, and $v_{z}=0.2\;\text{cm}$ located at the depths where profiles are to be extracted.

#### C.3 Monte Carlo calculation of ICF

The phsp files obtained just above the third scraper (level A‐A′ in Fig. 2) were used as input to the INSERT PHANTOM model. In this simulation, the particles sampled from the phsp file were transported through the last scraper or insert and in the water phantom. Using the voxel sizes mentioned above, dose/particle values as a function of depth were obtained along the central axis of the beam in phantom. Dose per incident particle $\left({\text{DP}}_{\max}\right)$ at $R_{100}$ is used as the beam output instead of dose per monitor unit.^(10)^ We chose this approach so that simulations with different numbers of histories could be compared directly. To avoid erroneous values for ${\text{DP}}_{\max}$, the data around $R_{100}$ were fit to a second‐order polynomial function, and the maximum value of the function was used for calculations. The statistical uncertainty of dose/particle at $R_{100}$ $\left(\Delta {\text{DP}}_{\max}\right)$ can be retrieved from the same output file as ${\text{DP}}_{\max}$. Typically, $40\times 10^{6}$ particles were simulated for this stage. Using this number of particles resulted in statistical uncertainties for ${\text{DP}}_{\max}$ of about 1% for large fields and 1% to 2% for small fields. The simulations were performed on a dual processor Dell Xeon 1.7 GHz machine. Simulation times are applicator‐ and insert size‐dependent. To achieve the above‐mentioned statistical uncertainties, the simulation times range between 1 h and 6 h. Since the vast majorities (>95%) of the inserts (cutouts) are being used with $10\times 10\;{\text{cm}}^{2}$, $15\times 15\;{\text{cm}}^{2}$, or $20\times 20\;{\text{cm}}^{2}$ electron applicators, we focused our investigation on these applicators only.

Insert correction factors were calculated using Monte Carlo simulation and the formalism described in TG‐25.^(15)^ For each applicator/insert the ${\text{DP}}_{\max}$ and its associated standard error $\Delta {\text{DP}}_{\max}$ were obtained from the output file. The ICFs and their associated standard errors were calculated using the following formulas:

(4) $$\text{ICF}(E,C_{s},I_{s})={\left(\frac{{\text{DP}}_{\text{max}}(E,C_{s},I_{s})}{{\text{DP}}_{\text{max}}(E,C_{s},I_{0})}\right)}_{\text{SSD}=\text{const}}$$

(5) $$\Delta \text{ICF}(E,C_{s},I_{s})={\left(\sqrt{{\left(\frac{\Delta {\text{DP}}_{\text{max}}(E,C_{s},I_{s})}{{\text{DP}}_{\text{max}}(E,C_{s},I_{s})}\right)}^{2}}+{\left(\frac{\Delta {\text{DP}}_{\text{max}}\left(E,C_{s},I_{o}\right)}{{\text{DP}}_{\text{max}}\left(E,C_{s},I_{o}\right)}\right)}^{2}\right)}_{\text{SSD}=\text{const}}$$

where ICF is for insert $I_{s}$, applicator $C_{s}$, and energy *E*; ΔICF is the standard error of ICF; ${\text{DP}}_{\max}$ is the maximum dose/particle on the central axis; $\Delta {\text{DP}}_{\max}$ is the standard error of the maximum dose output; $I_{s}$ is the insert size, $I_{o}$ is the insert size for the open applicator, $C_{s}$ is the applicator (cone) size, *E* is the nominal energy of the electron beam, and SSD is source‐to‐phantom distance.

#### C.4 Analytical modeling of ICFs

The calculated ICF data, as a function of the size of the insert for a specific applicator, was fit to the heat capacity model function of the following form:

(6) $$f(x)=a+bx+\frac{c}{x^{2}}$$

where *a*, *b*, and *c* are free parameters, and *x* is the parameter describing the insert. The values of *a*, *b*, *c* obtained from the fits are then used to evaluate the ICF for any insert size to be used in the clinic.

While Eq. (6) was adequate for most of the data, there were situations when a second‐ or third‐order polynomial function was used to fit the data:

(7) $$f(x)=\sum_{k=1}^{m}a_{k}x^{k-1}$$

Polynomials are more suited for large insert sizes where it was observed that ICF reaches a maximum around $7\times 7\;{\text{cm}}^{2}$ field size and then decreases slowly as the field size increases. For idealized clinical shapes characterized by two parameters (rectangle, ellipse, half‐ellipse) a 2D fit of the ICFs as a function of the ratio of the parameters was performed. First, the data were selected as a function of the ratio of the parameters a/b for a=const. Then the data were fit to a second‐order polynomial for each value of parameter *a*:

(8) $$f\left(\frac{a}{b}\right)={\left[g{\left(\frac{a}{b}\right)}^{2}+h\left(\frac{a}{b}\right)+k\right]}_{a=\text{const}}$$

where a/b is the ratio of the parameters describing the clinical shape, and *g*, *h*, and *k* are the coefficients of the polynomial function. Next, the coefficients *g*, *h*, and *k* as functions of the parameter *a* were fit to second‐order polynomial functions:

(9) $$g(a)=\alpha_{1}a^{2}+\alpha_{2}a+\alpha_{3}$$

(10) $$h(a)=\beta_{1}a^{2}+\beta_{2}a+\beta_{3}$$

(11) $$k(a)=\gamma_{1}a^{2}+\gamma_{2}a+\gamma_{3}$$

By combining Eq. (8) with Eqs. (9) to (11), the values of any rectangular, elliptical, or semi‐elliptical ICF can be obtained. For the trapeze inserts no attempt was made to fit the data to analytical functions; therefore, they are presented as tables.

In order to test the goodness‐of‐fit between the calculated and fitted data, the Kolmogorov–Smirnoff statistic (*D*) was calculated.^(24)^ In this work, a significance level of 0.1 (or a confidence level better than 90%) was considered acceptable for sample sizes between 7 and 15 points.

## III. RESULTS AND DISCUSSIONS

### A. Model validation

**Figure 4:**
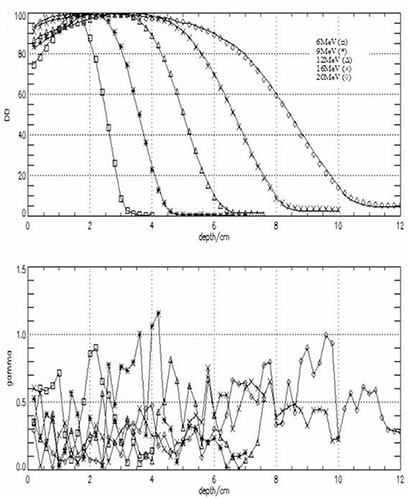
Measured (lines) and calculated (symbols) depth dose distribution (top panel) for a $10\times 10\;{\text{cm}}^{2}$ applicator, SSD=100 cm, all energies. The bottom panel shows the differences between measured and calculated values expressed as a γ index. The γ index was calculated using a dose difference ΔD=2.0% or a position difference Δd=2.0 mm criteria. Values of γ>1.00 indicate a point where the comparison criteria are not satisfied.

**Figure 5:**
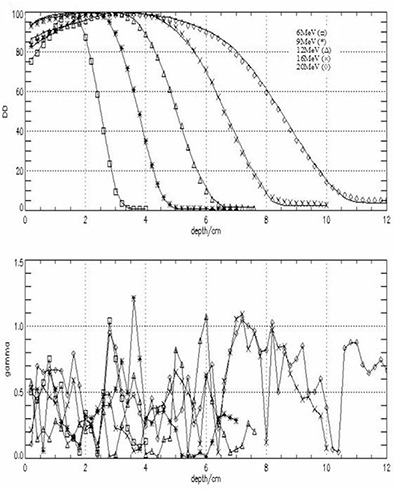
Measured (lines) and calculated (symbols) depth dose distribution (top panel) for a $20\times 20\;{\text{cm}}^{2}$ applicator, SSD=100 cm, all energies. The bottom panel shows the differences between measured and calculated values expressed as a γ index. The γ index was calculated using a dose difference ΔD=2.0% or a position difference Δd=2.0 mm criteria. Values of γ>1.00 indicate a point where the comparison criteria are not satisfied.

After fine‐tuning the primary electron energy for 6‐MeV to 20‐MeV electron beams, simulations were run for $10\times 10\;{\text{cm}}^{2}$ and $20\times 20\;{\text{cm}}^{2}$ applicators to validate the BEAM model. Figures 4 and 5 show measured and calculated DD distribution in water for $10\times 10\;{\text{cm}}^{2}$ and $20\times 20\;{\text{cm}}^{2}$ for 6 MeV to 20 MeV electron beams. The dimensions of the voxels along the central axis were as follows: radius r=1.0 cm and the height h=0.2 cm. These dimensions were found to be adequate for faster calculations without compromising the accuracy. Each curve was normalized to its own maximum value. Also shown in these figures are the γ index values for each measured and calculated pair. For both applicators good agreement between measured and calculated values was obtained. For the 10×10 applicator the γ index has a maximum value of 1.154 for a 9‐MeV electron beam, which corresponds to a maximum difference of about 2.3%; however, for the vast majority of the points, γ≤1.0. For the 20×20 applicator, the γ index has a maximum value of 1.21 for a 9‐MeV electron beam, which corresponds to a maximum difference of about 2.4%. The deviations appear to be larger for depths beyond $R_{50}$.

**Figure 6:**
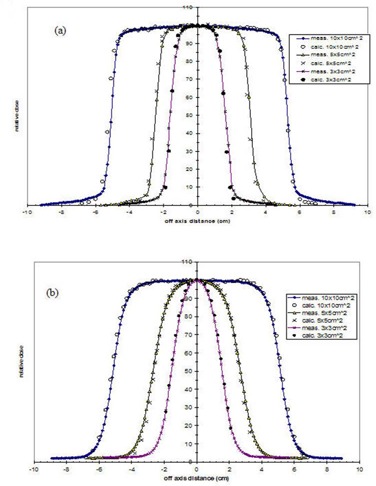
Calculated cross‐beam profiles for (a) 6‐MeV at depth d=1.5 cm and (b) 20‐MeV at depth d=2.0 cm electron beams versus measured data.

Calculated and measured cross‐beam profiles at depth of maximum dose, $R_{100}$, at 100 cm SSD for the 6‐MeV and 20‐MeV for $10\times 10\;{\text{cm}}^{2}$ applicator are shown in Fig. 6. The γ index is ≤1.0 for most of the profiles evaluated. For profiles evaluated at $R_{100}$, the γ index is <0.5, which translates to agreements better than 1.0% or 1.0 mm.

### B. Insert correction factors

A set of ICFs was measured and calculated for the idealized clinical shapes represented in Fig. 1. Table 2 shows the percentage difference between measured and calculated ICF values for 6 MeV to 20 MeV energies $10\times 10\;{\text{cm}}^{2}$, $15\times 15\;{\text{cm}}^{2}$, and $20\times 20\;{\text{cm}}^{2}$ applicators for the circular insert. Similar tables for the other inserts can be obtained from the author in PDF format. For certain shapes and the 15 applicator, only a small subset of ICFs was measured and compared with the calculated values as proof of model validity. The data are normalized to the open cone for which the insert is designed for all measured and calculated sets. Good agreement between calculated and measured ICF values was obtained. Deviations exceeding 2.0% are highlighted in gray. Maximum deviation observed was 4.12% for 6 MeV, $2\times 4\;{\text{cm}}^{2}$ in a $20\times 20\;{\text{cm}}^{2}$ applicator; however, for the vast majority of values, the agreement was better than 2.0%.

**Table 2:** Percentage difference between calculated and measured ICFs for the 6‐, 9‐, 12‐, 16‐, and 20‐MeV electron beams, $10\times 10\;{\text{cm}}^{2},15\times 15\;{\text{cm}}^{2}$, and $20\times 20\;{\text{cm}}^{2}$ applicators with a circular insert described by the radius r. The percentage differences are calculated using $\left[(({\text{ICF}}_{\text{calc}}-{\text{ICF}}_{\text{meas}})/{\text{ICF}}_{\text{meas}})\times 100\%\right]$. Values larger than 2.0% are shaded.

Circular insert		6 MeV	9 MeV	12 MeV	16 MeV	20 MeV
Applicator	*r* (cm)	%dif	%dif	%dif	%dif	%dif
10×10	5	0.21	–0.32	–0.38	–0.45	0.26
	4	0.33	0.32	–0.79	–1.73	0.82
	3	–0.92	–1.13	–0.17	–0.18	0.35
	2.5	–0.95	1.29	0.12	0.06	–0.12
	2	1.07	–0.19	–0.94	2.64	0.85
	1	0.61	–0.53	1.46	–0.12	0.94
15×15	7.5	–0.50	–0.04	–0.54	–0.41	0.20
	6	–0.62	–0.12	–0.67	–0.49	–0.41
	4	–0.47	–0.92	1.55	–0.69	–0.77
	3	–0.20	–0.60	0.18	–0.73	0.31
	2.5	0.67	0.65	–1.06	0.71	0.71
	2	–0.34	1.05	–0.86	0.20	0.81
	1	–2.57	0.60	–2.99	–0.96	1.40
20×20	10	0.00	0.24	0.10	–0.47	0.20
	7.5	–1.29	0.16	0.20	–0.32	–0.58
	6	–1.21	0.22	–0.40	0.18	–0.81
	5	0.29	0.18	–0.30	0.63	–0.07
	4	–2.23	–2.05	0.30	0.34	–0.90
	3	0.25	–0.56	0.82	0.89	–0.11
	2.5	–0.45	1.60	–0.29	–0.08	–0.52
	2	–0.12	2.17	–1.49	–0.06	0.59
	1	1.89	0.38	–2.32	1.60	1.44

General trends of the ICF values with energy and applicator size have been investigated and explained in previous studies.^(^ ^9^ ^–^ ^12^ ^)^ Insert correction factors have dose components from both direct and scattered particles. Direct electrons, which typically contribute 80% to 90% of the dose at $R_{100}$, are the primary influence on the shape of the ICF curve. For all energies, the direct electron component increases with field size until the insert size reaches electron range. The scattered electron component is slightly reduced for the smallest insert size because the insert blocks the electrons scattered from the upper scraper bars. As the insert size increases, the scattered electron component reaches a peak and is then slightly reduced for the largest insert sizes. The scattered component of the electron beam is strongly dependent on the shape of the insert, which makes it difficult to predict the ICFs.

From simple graphical representations, not presented here, the following trends can be confirmed: (a) for all energies and applicators, as the size of the insert decreases, the ICF first increases slowly to a maximum value located around *a* = 7.0 cm and then drops rapidly as the lower limit of the insert size is reached; (b) as the energy increases, the variation of ICF versus the size of the insert is reduced from 28% for a 6‐MeV electron beam to 3.6% for a 20‐MeV electron beam, $10\times 10\;{\text{cm}}^{2}$ applicator; and (c) as the applicator size increases, the variation of ICF is reduced for a particular energy, the effect being stronger for high‐energy electron beams (5.0 % for $10\times 10\;{\text{cm}}^{2}$ applicator to 2.6% for a $20\times 20\;{\text{cm}}^{2}$ applicator for the 20‐MeV electron beam, while for the 6‐MeV the values are virtually similar for all the applicators). As the energy of the electron increases, the particles scattered from the insert edges have enough energy to reach the point of measurement; thus the out‐scatter electrons are compensated by the in‐scatter electrons (from the insert edges) virtually maintaining constant particle fluence at the depth of measurement. The presence of the insert is similar to that of an inhomogeneity, and its effects on dose distribution are described by Khan.^(25)^

**Figure 7:**
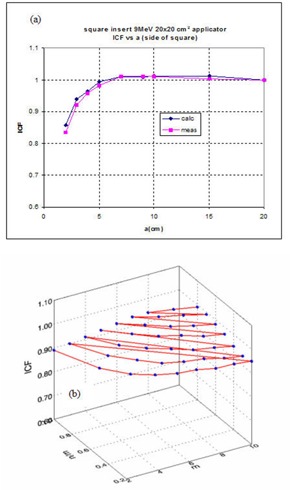
(a) ICF for square insert; 6 MeV, $20\times 20\;{\text{cm}}^{2}$ cone. (b) ICF for 12 MeV, $10\times 10\;{\text{cm}}^{2}$ cone elliptical insert.

A brief investigation of the calculated values shows that the ICFs as a function of the insert parameter(s) can be represented by analytical functions for the shapes considered in this study. Figures 7(a) and 7(b) show the variation of the ICF for the square and ellipse inserts as a function of their defining parameters. For inserts described by one parameter (i.e., square, circle, semicircle), either Eq. (6) or (7) was used to obtain the fitting parameters. The fits were performed using the least‐square fitting routines available on the Interactive Data Language (Research Data Inc, Boulder, CO). The goodness‐of‐fit between measured data and the values obtained from the Monte Carlo fitted data was tested using the K–S statistics, $D_{\text{KS}}$. The values of $D_{\text{KS}}$ relative to the expected values for a 95% confidence level that the two sets of data originate from the same distribution (the null hypothesis) are presented in tables similar to Table 3. The standard errors for measured ICFs are related to the precision of the measurements, which was found to be <0.5% as calculated using basic statistical formulas. The standard errors for Monte Carlo–calculated ICF are between 0.5% and 2.0%. Their upper limits are affected by the number of histories used for simulations.

**Table 3:** Parameters of the fits for the ICF as a function of the insert parameter for 6‐MeV, $10\times 10\;{\text{cm}}^{2\;\text{cone}}$, 100 cm SSD. The fitting coefficients are for square, circle, semi‐circle, rectangle, and ellipse.

Insert	Parameter	Function	Coefficients	$D_{\text{KS}}$	*p*
square	a→x	$y=u+vx+z/x^{2}$	u=1.0762036	0.143	0.001
			v=−0.0060768		
			z=−1.1105671		
circle	r→x	$y=u+vx+zx^{2}+wx^{3}$	u=0.4584	0.333	0.042
			v=0.4275		
			z=−0.1089		
			w=0.0091		
semi‐circle	r→x	$y=u+vx+zx^{2}+wx^{3}$	u=0.80401	0.2	0.012
			v=−0.07873		
			z=0.0546		
			w=−0.0065		
rectangle	a,a/b	$f\left(a/b\right)=g(a){\left(\frac{a}{b}\right)}^{2}+h(a)\left(\frac{a}{b}\right)+k(a)$	$\alpha_{1}=0.019$	0.167	0.107
*a* width		$g(a)=\alpha_{1}a^{2}+\alpha_{2}a+\alpha_{3}$	$\alpha_{2}=0.0318$		
*b* length		$h(a)=\beta_{1}a^{2}+\beta_{2}a+\beta_{3}$	$\alpha_{3}=-0.1881$		
		$k(a)=\gamma_{1}a^{2}+\gamma_{2}a+\gamma_{3}$	$\beta_{1}=-0.049$		
			$\beta_{2}=0.1376$		
			$\beta_{3}=-0.0556$		
			$\gamma_{1}=0.0085$		
			$\gamma_{2}=0.0572$		
			$\gamma_{3}=0.6663$		
ellipse	n,n/m	$f\left(\frac{n}{m}\right)=g(n){\left(\frac{n}{m}\right)}^{2}+h(n)\left(\frac{n}{m}\right)+k(n)$	$\alpha_{1}=0.0302$	0.08	0.001
*n* minor;		$g(n)=\alpha_{1}n^{2}+\alpha_{2}n+\alpha_{3}$	$\alpha_{2}=-0.3158$		
*m* major axis		$h(n)=\beta_{1}n^{2}+\beta_{2}n+\beta_{3}$	$\alpha_{3}=0.3765$		
		$k(n)=\gamma_{1}n^{2}+\gamma_{2}n+\gamma_{3}$	$\beta_{1}=-0.0461$		
			$\beta_{2}=-0.5304$		
			$\beta_{3}=-0.8815$		
			$\gamma_{1}=0.0058$		
			$\gamma_{2}=-0.084$		
			$\gamma_{3}=1.091$		

**Figure 8:**
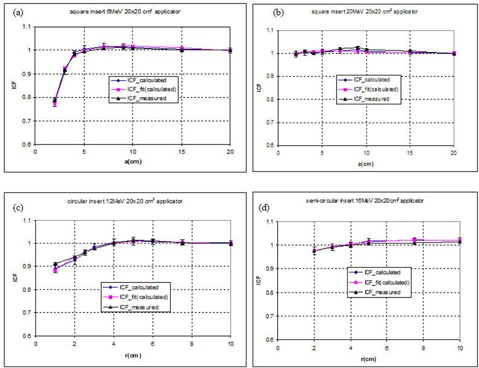
Calculated, fitted from calculated and measured values of the ICFs for the $20\times 20\;{\text{cm}}^{2}$ applicator. (a) 6‐MeV square insert; (b) 20‐MeV square insert; (c) 12‐MeV circular insert; and (d) 16‐MeV semi‐circular insert. The error bars represent the uncertainties in Monte Carlo–calculated values.

For all inserts described by one parameter (i.e., square, circle, and semicircle) the values of $D_{\text{KS}}$ obtained confirm the null hypothesis. Figures 8(a) to (d) show a comparison between measured, calculated, and fitted values of ICF for four combinations of energy, applicator, and insert shape. For each insert/applicator combination the function used to fit the Monte Carlo–calculated data and its associated coefficients is presented in tabular format. As an example, Table 3 shows the parameters obtained from fitting the 6‐MeV data.

**Figure 9:**
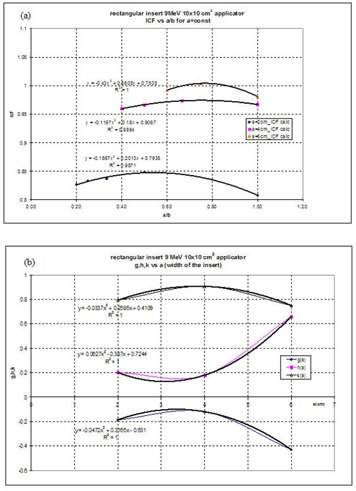
Data analysis for 9‐MeV electron beam, $10\times 10\;{\text{cm}}^{2}$ applicator, rectangular insert. (a) Calculated ICF values versus a/b ratios for a=const and (b) the parameters *g*(*a*), *h*(*a*), and *k*(*a*) versus *a* the side of the rectangle.

For inserts described by two parameters (i.e., ellipse, semi‐ellipse, rectangle), the modeling was performed in two steps as described in the Methods and Materials section. Figure 9(a)) shows the variation of rectangular ICFs versus ${\left(\frac{a}{b}\right)}_{a=\text{const}}$ for a 9‐MeV electron beam, $10\times 10\;{\text{cm}}^{2}$ applicator. Values of *g, h*, and *k* as a function of the parameter *a* are obtained. Figure 9(b) shows the dependence of *g h*, and *k* versus *a* for a 9‐MeV electron beam, a $10\times 10\;{\text{cm}}^{2}$ applicator, and rectangular inserts. Using Eqs. (9) to (11), the coefficients for fitting *g, h*, and *k* as a function of the parameter *a* are obtained. Finally, the whole matrix of ICF values could be obtained using Eq. (8) and the nine fitted parameters. The values of the coefficients are then recorded in tables similar to Table 3. Additional tables for inserts and applicators in PDF format are available from the author.

To simplify the calculations of the ICFs by using the fitting coefficients, a simple routine was written using Microsoft Visual $C^{++}$. The user supplies the energy, insert shape, applicator size, and depth of prescription. The routine calculates the ICF and prints a %DD graph for the insert considered. The presence of the %DD printout will help the radiation oncologist and medical physicist verify the validity of selected energy and insert for a particular case.

## IV. SUMMARY AND CONCLUSIONS

The purposes of this work were to investigate the use of Monte Carlo simulation methods to predict the output factor for various shape inserts used in a clinical setting, and the possibility of analytical modeling of output factors for clinical electron beams. The EGS4/BEAM user code provided the ability to do Monte Carlo simulations of all the relevant components of the accelerator treatment head and produce phase space files for the vast majority of energy/applicator combinations available in the clinic. Once accurate phase space files were obtained, dose distributions for a variety of inserts were calculated.

The agreement between measured and calculated dose distribution in water is within 2.0% or 2.0 mm for percentage depth‐dose and cross‐beam profiles for all energies. Insert correction factors, measured using a parallel‐plate ionization chamber, were compared with Monte Carlo–calculated values. Insert correction factors could be predicted with an accuracy of 2.0% for most of the shapes considered in this study. There are a few exceptions where differences of up to 4.0% were recorded. Larger variations are seen for very small inserts, and the discrepancies can be attributed to our inability to measure the data correctly. Most values outside the 2.0% criterion were observed for small rectangular inserts with large $\frac{a}{b}$ ratios. The shape affects the output more than the size of the insert.

Analytical functions can be used to model the ICF dependence versus the size of the insert for most of the shapes considered; therefore, ICF tables can be produced for clinical use.

To calculate the complete set of data for this project, 10 commissioning simulations, 15 ABOVE_INSERT, and 835 (167 calculations/energy for 5 energies) simulations for ICF were run. All variance reduction techniques available in BEAM user code were used to reduce simulation time. The overall running time for the simulations in this study is evaluated at 250 CPU days on a Dell Xeon 1.7 GHz PC running Linux OS. Depending on the complexity of the insert, the simulation of a new insert will take between 1 h and 6 h to complete on the above‐mentioned equipment.

If the insert is small and highly irregular (sharp corners, distance between measurement point and edge of insert $\leq R_{p}$), the functions presented in Table 3 or similar may not provide accurate results; therefore, individual simulation and/or measurements should be performed.

All calculations presented in this study were performed for an SSD of 100.0 cm. The ICF values were found to be accurate to within ±2.0% for SSDs up to 105.0 cm. For larger SSD values (110 cm to 115 cm) a similar study will have to be performed.

Over a period of 18 months we have found that for about 99% of the clinically used inserts, the methods described in this study could be used to obtain the ICF. These findings have resulted in tremendous labor savings for the clinical medical physicist.
